# Epidemiological Characterization and Risk Factors of Allergic Rhinitis in the General Population in Guangzhou City in China

**DOI:** 10.1371/journal.pone.0114950

**Published:** 2014-12-16

**Authors:** Chun Wei Li, De Hua Chen, Jia Tao Zhong, Zhi Bin Lin, Hua Peng, Han Gui Lu, Yan Yang, Jia Yin, Tian Ying Li

**Affiliations:** 1 Department of Otolaryngology, National University of Singapore, National University Health System, Singapore; 2 Department of Allergy, The First Affiliated Hospital of Sun Yat-Sen University, Guangzhou, China; 3 Department of Otorhinolaryngology, The First Affiliated Hospital of Sun Yat-Sen University, Guangzhou, China; 4 Department of Nutrition, School of Public Health, Sun Yat-Sen University, Guangzhou, China; 5 Department of Allergy, Peking Union Medical College Hospital, Chinese Academy of Medical Sciences & Peking Union Medical College, Beijing, China; University of Texas Health Science Center San Antonio Texas, United States of America

## Abstract

The prevalence of allergic rhinitis (AR) in China has increased with an apparent geographic variation. The current study aims to investigate the AR prevalence/classification, diagnosis/treatment conditions, trigger factors, and risk factors in the general population of Guangzhou, the third biggest city in China. A cross-sectional survey was performed in the citizens in Guangzhou from December 2009 to March 2010 by using a stratified multistage cluster sampling method. All subjects were asked to complete a comprehensive questionnaire via a face to face interview. A total of 9,899 questionnaires were valid. The prevalence rate of AR in the general population of Guangzhou was 6.24%, with a significant higher prevalence in urban area (8.32%) versus rural area (3.43%). Among the AR subjects, most (87%) were diagnosed with intermittent AR and 87% suffered from moderate-severe symptoms. High percentages of the AR patients did not have previously physician-based diagnosis (34%) or specific medical treatment (55%). Morning time, winter season, and cold air were the most common trigger factors of AR. Family history of AR, current living place, living place during babyhood, smoking, home renovation, and pet ownership were the significant risk factors associated with AR prevalence in the population. The study demonstrated comprehensive epidemiological and clinical information about the AR in Guangzhou population. Change of living environment and lifestyles had strong impacts on the prevalence of AR. Public health policies should help the patients benefit from a proper diagnosis/treatment and specifically target the local risk factors, in order to control the AR incidence.

## Introduction

Allergic rhinitis (AR) is a chronic inflammatory disease of the nasal mucosa. An increase of release of inflammatory mediators and cytokines is significant in AR which is triggered by allergens and is mediated by antibody immunoglobulin E (IgE). The prevalence of AR has increased substantially in recent decades in China and it imposes a heavy healthy and socioeconomic burden on the patients. Although several cross-sectional population-based studies have been performed to analyze the prevalence of AR in multiple major cities [1], the characteristics of the AR trigger factors and the associated risk factors have been less reported. In addition, large variation existed in the epidemiological data regarding the prevalence rate of AR in different cities, ranging from the lowest in Beijing (8.7%) to the highest in Urumqi (24.1%) [1]. The regional variation is attributed to the huge diversity and difference of population size, environmental and socioeconomic conditions. Thus, in-depth research is needed to evaluate the epidemiological characters/clinical information of AR and the risk factors of AR based on different geographical area.

To gain the prevalence, the distribution and the health economic information in Chinese general population about the allergic diseases (involving AR, asthma, food allergies, atopic dermatitis, urticaria), and to formulate the prevention interventions of them which can adapt the different income levels in China, a national wide project called “National Epidemiology Study of Asthma and Allergy in China (NESAAC)” was established in 2007 by the Chinese Ministry of Health. The project is an epidemiological survey, leading by Chinese Academy of Medical Sciences and Peking Union Medical College Hospital, and participated by a number of medical units (including 19 centers in 18 cities, Guangzhou is one of them). Guangzhou is the third biggest city in China, with a population of around 8.3 million citizens and highest gross domestic product in the southern part of China. This study investigated the prevalence/classification of AR, diagnosis/treatment conditions, triggers factors, and associated risk factors of AR in the general population of Guangzhou city. The analysis of comprehensive parameters will estimate a more accurate prevalence rate of AR, describe the clinically epidemiological information of AR, and determine the risk factors of AR in the local population, in order to provide useful information for clinicians and health policymakers making specific strategies to control the AR prevalence.

## Methods

### 1 Ethics statement

The study was approved by the ethics committee of the First Affiliated Medical Hospital of Sun Yat-Sen University in Guangzhou, China. Before doing the questionnaire, the adult participants had signed the informed consent, while the consent document for the children aged less than 18 years had been signed by their parents or guardians. The questionnaire forms for those younger people aged less than 18 years old were completed by a parent or a guardian of the child.

### 2 The study population and sampling method

**Figure 1:**
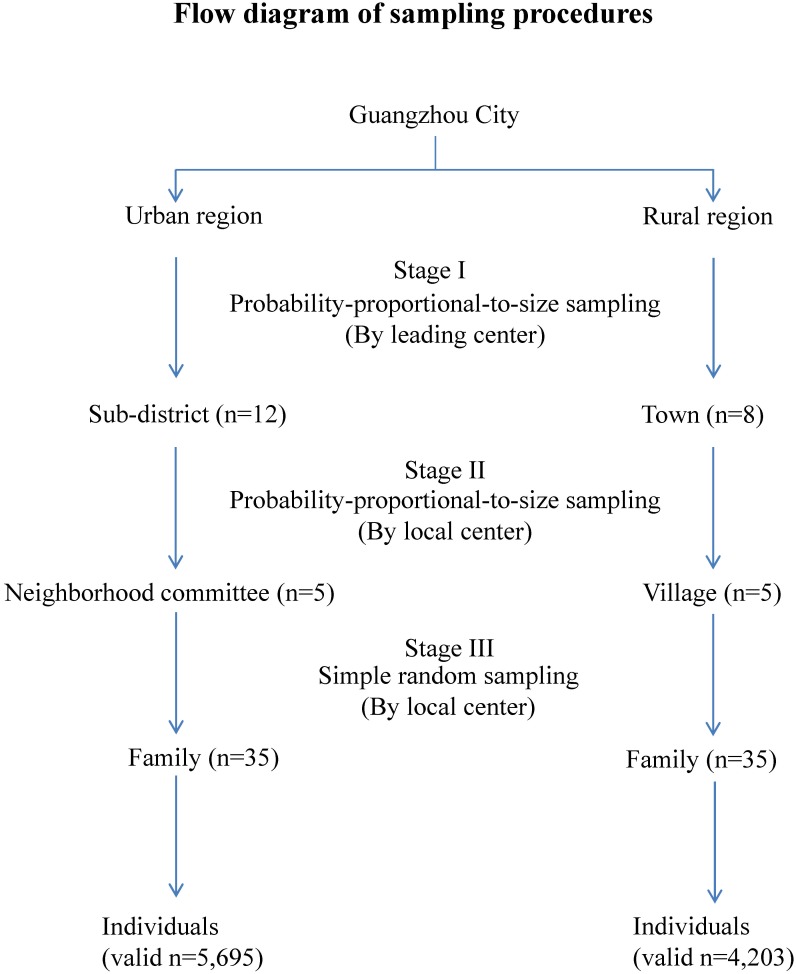
Flow diagram for sampling methods and procedures in the study.

A cross-sectional survey was conducted among the general population in Guangzhou city during Dec. 2009 to Mar. 2010, using a stratified multistage sampling method (Fig. 1). The population from Guangzhou city was separated into two independent strata based on the places of residence, i.e., urban and rural areas. Probability-proportional-to-size (PPS) sampling, in which the selection probability for each unit was set to be proportional to its population size, was utilized to sample 12 sub-districts from urban region and 8 towns from rural region, respectively. This stage I sampling procedure was performed by the leading center of the NESAAC project. In Stage II, 5 neighborhood committees from each sub-district and 5 villages from each town were selected by using PPS sampling method. In Stage III, 35 families from each neighborhood committee or village were chosen by using a simple random sampling method. The individuals in each selected family, who registered more than 6 months, were enrolled in the survey. The sampling process of Stage II and III was conducted by the participating local center. All the sample units of each stage were encoded confidentially and then randomly sampled by the computer. The estimated sample size was about 10,000, including 6000 in urban and 4,000 in rural areas. The gender and age of the sampled respondents was not limited.

### 3 Questionnaire design

The standardized questionnaire was derived from the well-documented questionnaires used by the International Study of Asthma and Allergies in Childhood (ISAAC) [2]. The criteria used to diagnose AR were based on the document of Allergic Rhinitis and its Impact on Asthma (ARIA) 2001 [3]. Each questionnaire contains 76 questions, including demographic information, general situation (e.g., marriage, occupation, and education level), screening questionnaire of AR (e.g., symptoms, timing of symptom appearance, and history of diagnosis), professional questionnaire of AR (e.g., age at onset of symptom, frequency of symptom, disease severity, trigger factors of symptom, and treatment condition), questionnaire of AR risk factors (e.g., living area, income, history of smoking, home renovation, breastfeeding, computer usage, and family history). Those screening questionnaires which were not filled in the information about typical AR symptoms (used for AR diagnosis) were considered invalid.

### 4 Data collection

The study was conducted by the department of otolaryngology & department of allergy, The First Affiliated Hospital of Sun Yat-Sen University in Guangzhou, China. The survey was administered by the ENT doctors and well-trained graduate students from medical school via a face-to-face interview. Under the coordination of the local government, all the residents who were sampled were recruited to the community centers by staff from the neighborhood or village committees. For those residents who could not come to the center, the interviewers would visit their home to complete the survey. Before doing the questionnaire, the adult participants had signed the informed consent, while the consent document for the children aged less than 18 years had been signed by their parents or guardians. The questionnaire forms for those younger people aged less than 18 years old were completed by a parent or a guardian of the child. All the respondents need to fill out the screening questionnaire and the risk factor questionnaire; while the professional questionnaire of AR was completed only by patients. According to the diagnostic criteria of AR in the ARIA 2001 Guideline [3], the ENT specialists verified the screening questionnaires and made the diagnosis based on the typical AR symptoms (e.g., nasal itching, sneezing, nasal obstruction and rhinorrhea) within the last 12 months. The respondents with AR were interviewed by the ENT doctors and were asked to complete the professional questionnaire. Intermittent AR was determined when the symptoms occur <4 days per week or <4 consecutive weeks/year; while persistent AR was determined when symptoms last >4 days/week or >4 consecutive weeks/year. The symptoms are considered mild with normal sleep, no impairment of daily activities, no impairment of work or school, and if symptoms are not troublesome. The symptoms are considered moderate to severe with sleep disturbance, impairment of daily activities, and impairment of school or work.

### 5 Statistical analysis

All questionnaires were verified and invalid questionnaires were removed. Database was established with EpiData3.1 by the staffs. All statistical analyses were performed by a professional statistician using SPSS 18.0 software (SPSS Inc., Chicago, IL). The chi-square test was used to analyze the AR prevalence in the categories of gender, age group, and living areas; and to analyze the relationship between AR classification and diagnosis/treatment conditions. The Generalized Estimating Equations (GEE) which allows for dealing with correlated observations were used to analyze the risk factors. In the GEE procedure, family was considered subject variable and the people was within-subject variable; binary logistic regression was selected as the analytic model. A *p*-value less than 0.05 was considered as statistically significant.

## Results

### 1 Demographics of the survey subjects

A total number of 10,153 questionnaires were assigned, and the response rate was 99.8%. There were 9,899 valid screening questionnaires (effective rate 97.7%) for the study. Of all subjects, 5,266 (53%) were male and 4,632 (47%) female. 5,696 subjects (58%) lived in urban area while 4,203 (42%) lived in rural area.

### 2 Prevalence of AR

**Figure 2:**
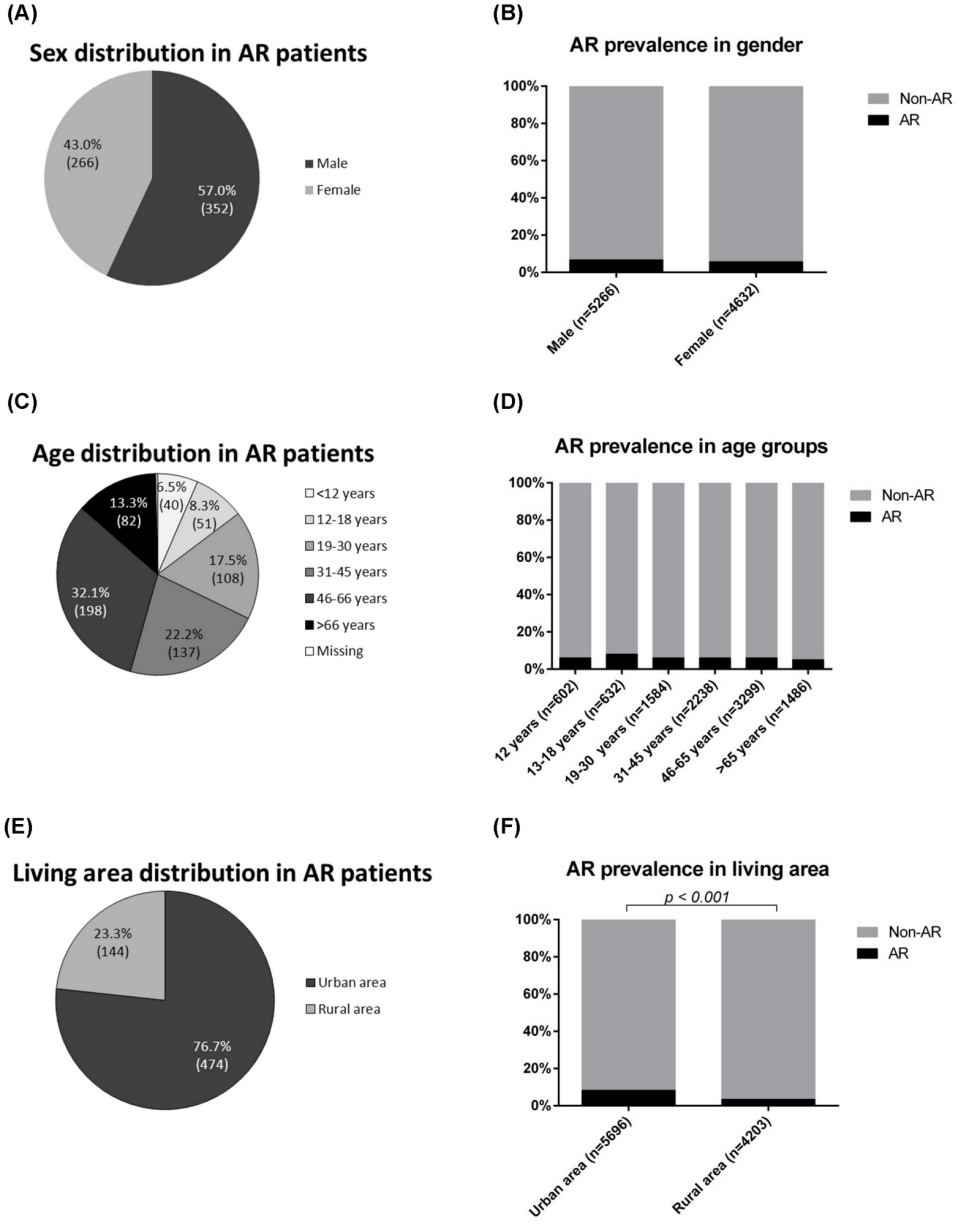
Epidemiological characteristics of AR. Sex distribution (A), age distribution (C), and living area distribution (E) of AR patients. The prevalence of AR in different age groups (B), in sex (D), and in different living areas (F). Exact number was indicated in the brackets in pie charts. Chi-square test was used to analyze the difference of the prevalence rate within each demographic group. A *p*-value below 0.05 was considered statistically significant.

Based on the screening questionnaire of AR, the information of AR-related symptoms within pass 12 months was noted and the prevalence rate of AR in the population was 6.2% (618/9,899). Among the 618 subjects with AR, 352 (57%) were male and 266 (43%) female (**Fig. 2A**). There was no significant difference in AR prevalence between males (352/5,266, 6.7%) and females (266/4,632, 5.7%) (**Fig. 2B**). The age distribution of the AR subjects was: 6.5% aged less than 12; 8.3% aged from 12 to 18; 17.5% aged from 19 to 30; 22.2% aged from 31 to 45; 32.1% aged from 46 to 66; 13.3% aged more than 66 (**Fig. 2C**). The prevalence of AR in the different age groups did not have statistical difference: 6.6% in subjects aged less than 12; 8.1% in subjects aged from 12 to 18; 6.8% in subjects aged from 19–30; 6.1% in subjects from 31–45; 6.0% in subjects from 46–66; and 5.5% in subjects aged more than 66 (**Fig. 2D**). In AR subjects, 77% (474/618) stayed in urban districts while 23% (144/618) stayed in rural districts (**Fig. 2E**). The AR prevalence rate was significantly higher in the people living in urban area (474/5,696, 8.3%) than those in rural area (144/4,203, 3.4%) (**Fig. 2F**).

### 3 AR classification and trigger factors

**Figure 3:**
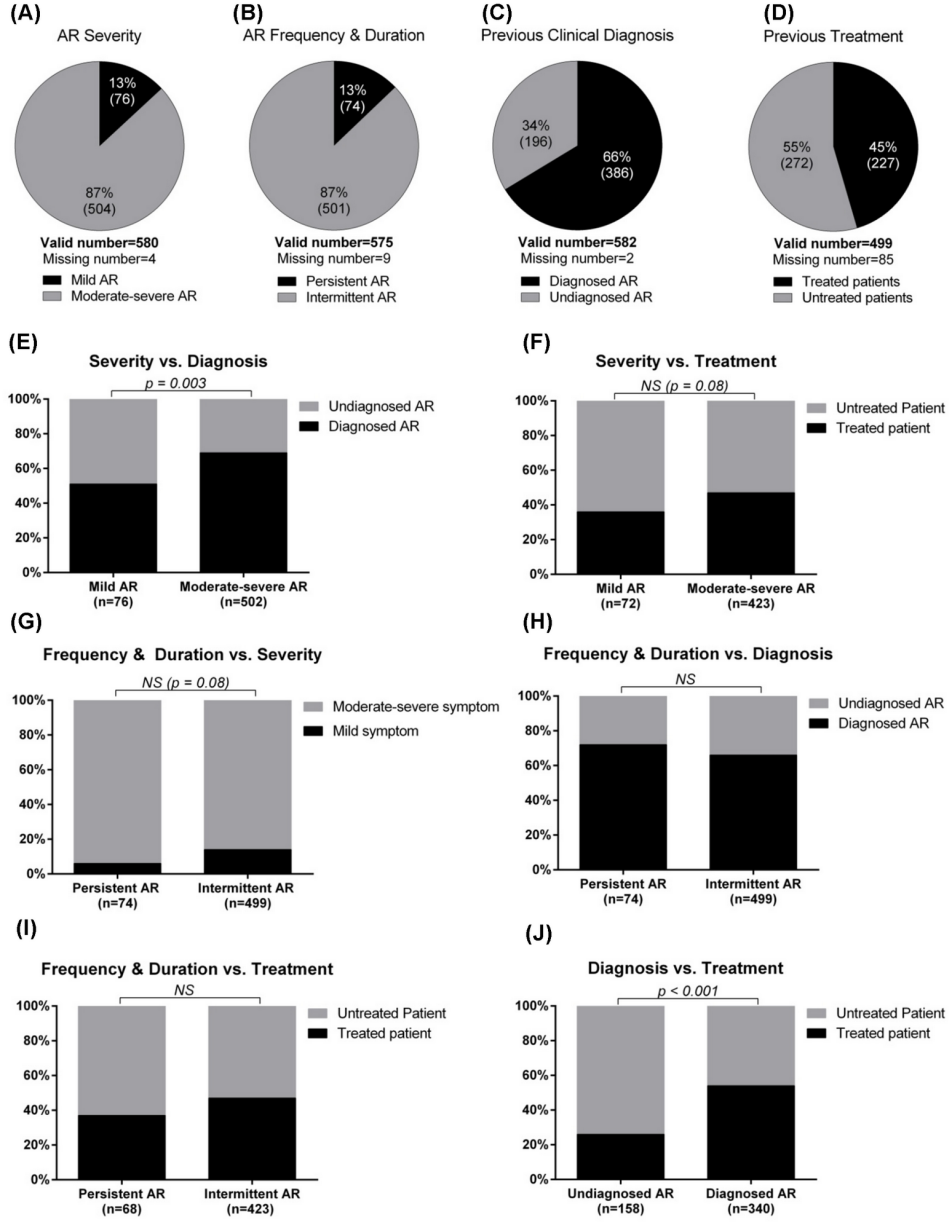
Clinical information of AR. Classification of AR based on the symptom severity (A) and frequency & duration (B); conditions of previous clinical diagnosis (C) and previous medical treatment (D). Relationships between AR classification and diagnosis/treatment status were analyzed by chi-square test: symptom severity and diagnosis (E), symptom severity and treatment (F), symptom severity and symptom frequency/duration (G), symptom frequency/duration and diagnosis (H), symptom frequency/duration and treatment (I), and diagnosis and treatment (J). A *p*-value below 0.05 was considered statistically significant.

The AR subjects (n = 618) were asked to filled in the professional questionnaires and 34 forms were not responded, leaving 584 questionnaires were validated. The data provided information about the classification of AR, previous diagnosis/medication and the relevant trigger factors of AR. Based on the symptom severity, 87% of the patients were grouped into moderate-severe AR, and 13% into mild AR (**Fig. 3A**). According to the new ARIA guideline considering the frequency and duration of the symptoms [3], 87% of AR patients were classified as intermittent and 13% were persistent (**Fig. 3B**). Sixty-six percent patients reported having a previous doctor diagnosis of AR (**Fig. 3C**), while only 45% claimed receiving treatment before (**Fig. 3D**).

The relationship between AR classification and diagnosis/treatment status was further analyzed. The patients with moderate-severe symptoms (69%, 345/502) had a significant higher chance to have a previous physician-based diagnosis of AR than those with mild symptoms (51%, 39/76) (**Fig. 3E**); these patients with moderate-severe symptoms (47%, 200/423) were also more likely to receive treatment before than the mild ones (36%, 26/72), and the difference just fell short of significance (*p* = 0.08) (**Fig. 3F**). When the two classifications of AR were cross-tabulated, it appeared that more patients with persistent AR (93%) had moderate-severe symptoms than those with intermittent AR (86%), approaching borderline significance (*p* = 0.08) (**Fig. 3G**). The proportion of previous clinical diagnosed AR was 65% and 72% in patients identified as intermittent and persistent, respectively (**Fig. 3H**); while the proportion of patients with previous treatment was 46% and 37% in the intermittent and persistent AR, respectively (**Fig. 3I**). As expected, the rate of previous medication was much higher in subjects with a previous diagnosis of AR (54%, 185/340) than those undiagnosed (26%, 41/158) (**Fig. 3J**).

**Figure 4:**
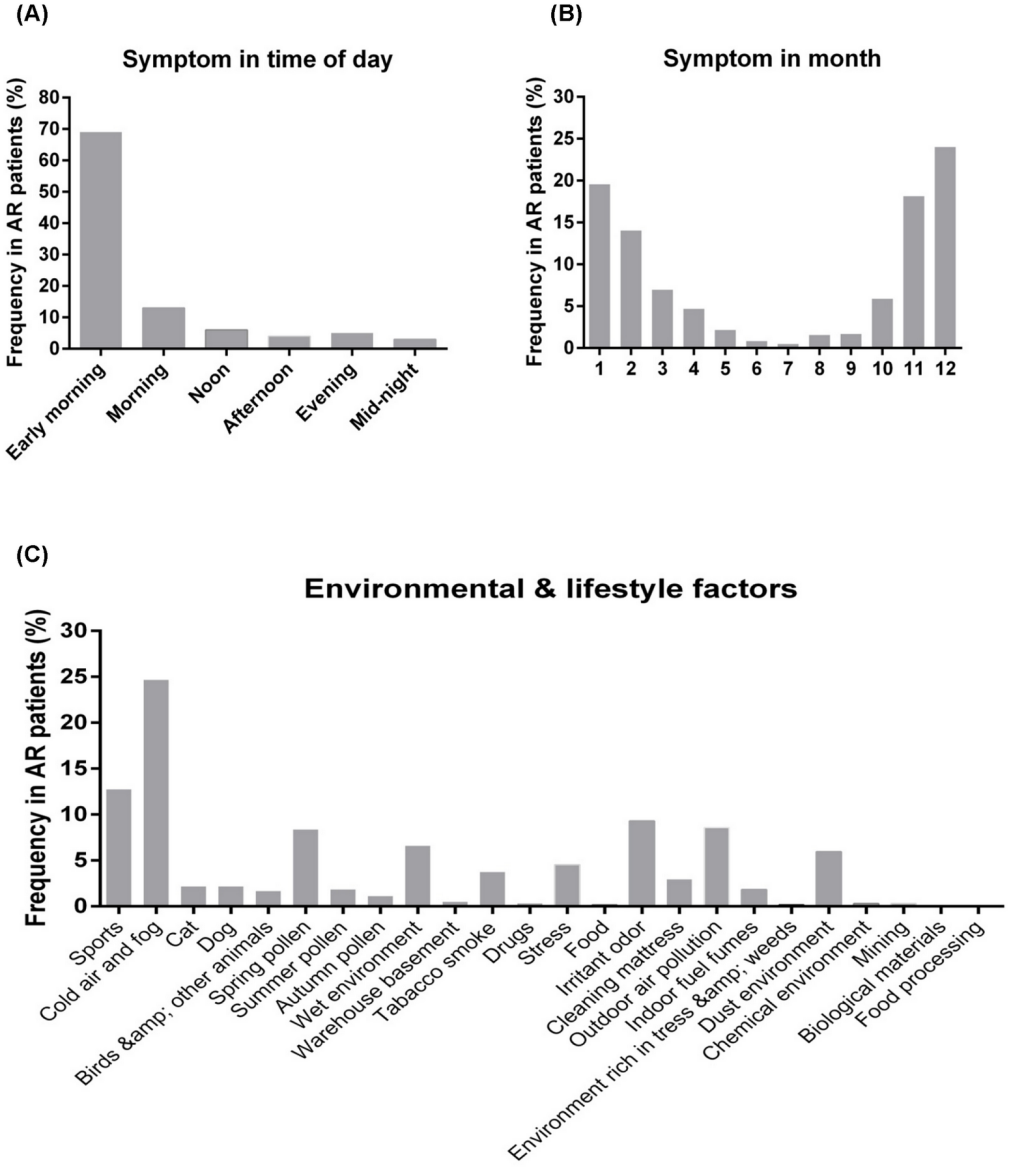
Trigger factors of AR were shown in three categories: time of day (A), season (B), and common 24 environmental/lifestyle factors (C). Bar charts showed the percentage of the selection frequency of individual factor under each category.

Trigger factors which contribute to exacerbation of the symptoms were analyzed. The patients were asked whether the symptoms vary by time of day or season, and exposure to environmental and lifestyle factors. The majority (69%) of the patients indicated that the most severe symptom episodes occurred in the early morning, and the symptom severity gradually reduced over time until midnight (**Fig. 4A**). Out of the total patients, 287 (49%) of them complained that the symptom severity differed in seasons. The symptom became more severe in most of the patients (75%) in winter (November, December, January, and February), followed by 12% in spring (March and April), 7% in summer (from May to September), and 6% in autumn (October) (**Fig. 4B**). There were still 259 patients (44%) who reported the symptom severity was not influent by seasons and 38 patients (7%) reported not clear for seasonal factor. Patients were required to select at least one environmental/lifestyle factor (maximum 5 factors) which would lead to symptom more severe. Twenty-four environmental/lifestyle factors were evaluated (**Fig. 4C**). 452 patients (77%) claimed that the symptom appeared more severe by exposure to these factors. The top inducing factors were cold air and fog (25% in selection frequency); other major factors like sports, spring pollens, irritation odor, and outdoor air pollution accounted for 13%, 8%, 9%, and 8% in total selection, respectively. Patients also complained some factors like wet environment (6%), dust environment (6%), tobacco smoke (4%), stress (4%), and cleaning mattress (3%) would induce more serious allergic symptoms (**Fig. 4C**).

### 4 Risk factors of AR

To evaluate the AR risk factors, 9,853 out of 9,899 questionnaires were successfully completed. Fifteen relevant risk factors were analyzed, including biological condition (age, breast feeding, family history, immunization in early childhood, and parasites infection in childhood); environment/lifestyle (current living place, living place from birth to 2 years old, history of smoking, hair coloring, home renovation, frequency of computer usage, history of owing a pet, and history of kindergarten); socioeconomic status (monthly income and car ownership). Three factors had too many missing values (parasites infection in childhood, missing value = 2,737; kindergarten, missing value = 1,196; immunization in early childhood, missing value = 1,561), which were considered leading to large errors, and they were excluded in the analysis.

Based on a binary logistic regression analysis for the remaining 12 factors, the results showed that 7 relevant factors were statistically significant (*p*<0.05), including family history of AR, living place during babyhood, smoking, home renovation, computer usage, and pet ownership (**Table 1**). Among these risk factors, family history of AR demonstrates the highest odd ratio (OR = 3.51). People living in urban area was more associated with AR prevalence than those in rural area (OR = 1.91). The risk of AR increased progressively in the people who lived in towns (OR = 1.67), and medium to large-sized cities (OR = 2.39) during babyhood as compared to those who lived in villages. People who have smoked (OR = 1.44), experienced home renovation (OR = 1.39), or owned a pet (OR = 1.35) also had a significant risk to AR incidence. Interestingly, people using computer occasionally (OR = 1.45), less than 2 hs daily (OR = 1.46), or 2–4 hs daily (OR = 1.58) a higher risk of AR than those never used computer; while higher frequency of computer usage (e.g., more than 5 hs daily) was not associated with the risk of AR incidence.

**Table 1:** Binary logistic regression of risk factors for AR.

Variables	*p*-value	OR (95% CI)
**Age (continue variable)**	0.29	0.99 (0.99, 1.00)
**Family history of AR**		
•No		1
•Yes	<0.001	3.51 (2.65, 4.64)
**Breastfeeding**		
•<6 months		1
•≥6 months	0.31	0.89 (0.73, 1.11)
**Living place (currently)**		
•Rural area		1
•Urban area	<0.001	1.91 (1.37, 2.68)
**Living place (from birth to 2 years old)**		
•Village		1
•Town	0.14	1.67 (1.11, 2.52)
•City	<0.001	2.39 (1.63, 3.54)
**Smoking**		
•Non-smoker		1
•Smoker	0.007	1.44 (1.10, 1.88)
**Hair coloring**		
•No		1
•Yes	0.61	1.13 (0.71, 1.79)
**Home renovation**		
•No		1
•Yes	0.02	1.39 (1.06, 1.81)
**Frequency of computer usage**		
•Never use		1
•Use occasionally	0.008	1.45 (1.10, 1.91)
•Use <2 hs daily	0.01	1.46 (1.10, 1.93)
•Use 2–4 hs daily	0.006	1.58 (1.14, 2.19)
•Use 5–8 hs daily	0.58	0.81 (0.38, 1.72)
•Use >8 hs daily	0.55	0.56 (0.08, 3.79)
**Pet ownership**		
•No		1
•Yes	0.03	1.35 (1.03, 1.77)
**Income**		
•Low income: < RMB 1,000		1
•Middle income: RMB 1,001–3,000	0.45	1.13 (0.83, 1.53)
•High income: > RMB 3,000	0.89	1.03 (0.65, 1.65)
**Car ownership**		
•No		1
•Yes	0.85	1.05 (0.64, 1.72)

OR: odd ratio; CI, confidence interval. Statistically significant at *p*<0.05.

## Discussion

There has been an increase trend of AR prevalence in both developed and developing countries in recent decades [4], [5]. The current study for the first time reported the prevalence and classification of AR as well as its trigger and risk factors in the general population of Guangzhou, the third biggest city in China. The prevalence rate of AR was 6.2% with significantly higher prevalence in urban areas (8.3%) than in rural areas (3.4%). These results indicate that the prevalence of AR is likely to increase because of rapid urbanization and changing environmental factors in the Mainland China. The most common aeroallergen in China is house dust mites and the prevalence of sensitization to mites is very high in AR patients [6]. Guangzhou locates in the southern coast regions and the environment is warm and humid, which is suitable for growth of the mites. People in urban areas are considered to experience more indoor activities, both at working and home places, where the ventilation is usually not good, leading to a higher chance to expose a high concentration of house dust mite. In addition to the indoor environment, the outdoor places of urban areas are affected by air pollution, which consists of a complex mixture of compounds, like SO_2,_ NO_2_, and PM2.5 or PM10 particles. These irritant pollutants are believed to increase the susceptibility of airway mucosa to allergen attack and development of allergic reactions [7]–[9]. Therefore, the above situations may explain the greater prevalence of AR in urban districts.

The prevalence rate reported in this study is lower than the previous report which showed a 14.1% AR prevalence in Guangzhou [1]. Zhang et al. used telephone interview to collect the data, although this method requires less time and money, it has some limitations on the low response rate and selection bias. Importantly, since telephone coverage rates of rural areas were low in China, the AR prevalence rate in Zhang et al.’s study was considered to be overestimated in the general population. The prevalence obtained by our study was also lower than the self-reported prevalence (19%) in a telephone survey carried out in 6 European countries [10]. The difference may be due to the different sample collection methods and the socioeconomic and environmental conditions.

The previous survey reported the proportion of persistent and intermittent AR in Guangzhou was 19% and 81%, respectively [1]. Similarly, our study showed 13% of the subjects were identified as persistent AR while 87% as intermittent AR, indicating intermittent type accounts for the majority of AR. Compared to the percentage of persistent AR (24.6–38.7%) in northern cities of China, the proportion of persistent AR (15.4–21.2%) found in southern cities (like Guangzhou) was lower [1]. Similar trend was also reported in northern European countries (24.8–53.8%) versus southern countries (21.0–32.4%), although the proportion of persistent AR subjects was higher in Europe than in China [10]. The variation of persistent AR prevalence between northern and southern parts of China or Europe may be mainly attributed to the climate condition and sensitization to allergens. This is because: 1) the winter time is usually longer in northern places and cold air is considered to be a trigger factor for AR; 2) persistent AR patients are relatively more often sensitized to pollens, which are produced by the specific seasonal plants grew in northern areas.

Our results showed that the previous diagnosis (66%) and medication (45%) rates were low in AR patients. Although the subjects with persistent AR experienced more severe discomfort, only 72% of these patients had a physician-based diagnosis and 37% received treatment before. In addition, among the patients who had been diagnosed by clinicians, just 54% of them received specific treatment. The lower diagnosis rate may be attributed to the strained medical resources and the busy lifestyle in the big city in China, making patients reluctant or no time to visit clinics. The imbalance in the allocation of medical resources in some province (e.g., Guangdong), where the patients from small to medium sized cities would prefer seeking medical specialists in the capital city (e.g., Guangzhou), leads to a shortage of medical resources in those big cities. With regard to the lower treatment rate, we consider the patients may concern the cost of the treatment, since both pharmacological therapy and specific immunotherapy are not cheap in China and it usually requires a long course of treatment, leading to a decline in the rate of treatment. Therefore, disseminating information about the disease treatment, optimization of the medical resources and introduction of the therapies in medical insurance scheme supported by government may improve the diagnosis and treatment rates.

Based on the analysis of AR trigger factors, cold air or cold weather appears the top 1 inducing factor. In winter, the level of the dust and contaminants is elevated, and the indoor ventilation is poor (usually doors and windows are closed), leading to the increase of opportunity to contact allergens. On the other hand, the big variation of temperature in cold weather or cold environment would affect the stability of the parasympathetic nervous system and the dry air would also increase the vascular permeability, resulting in the hypersensitivity of the topical nasal mucosa. Other trigger factors like spring pollen, wet environment and dust environment are related to a high chance of allergen (pollen and house dust mite) exposure; while sports, irritant odor and outdoor air pollution are associated with the stimulation of local mucosa response.

The highest risk factor for AR in general population in Guangzhou is the family history, i.e. genetic factor. It is in line with many epidemiological investigations performed in both Chinese and Caucasians which found that AR has a significant familial aggregation [11]. The chromosomal association studies identified the most repeated regions were involved in chromosomes 2, 3, 4, and 9 [12]; while single nucleotide polymorphism studies determined the polymorphisms in genes from different functional groups contributing to AR pathogenesis, such as chemokines/receptors, interleukins/receptors, and leukotrienes/receptors [12]. Recent study reported a novel susceptibility locus 14q11.2 for AR in Han Chinese population [13].

Accumulating evidence has confirmed that both genetic and environmental factors play important roles in the development of AR. A sharp rise of AR prevalence in recent years could be not only explained by genetic background, but also environmental factors, especially the rapid urbanization and the widespread of “westernization” lifestyles in China can be considered an essential reason. Our study found a cluster of “modern” lifestyle factors (e.g., living in urban area, living in urban city during babyhood, smoking, computer use, home renovation, and pet ownership) were significant risk factors of AR in Guangzhou general population, indicating these may be unhealthy habits with regard to the incidence of AR. Among these non-genetic aspects, living place during birth to 2 years old is the most significant factor for AR. Compared to living in village, the risk of developing AR progressively increases in the people who staying in town and in urban city. Similar results were reported that the farming environment in childhood provided a protective effect in adult against the development of allergic diseases [14]. This phenomenon is in agreement with the “hygiene hypothesis” which states that a lack of exposure to infectious agents during early childhood would increase the susceptibility to allergic diseases [15]. In village, the baby is usually growing in a larger family and in a relatively lower standard of living, causing a higher chance to expose diversified microbial (especially those microbiota) and the spread of infections by person to person. Infectious pathogens elicit a Th1 mediated immune response, which could reduce Th2 response, in turn may lead to more allergic response or the impairment of immune tolerance [16]. In town or urban city, the economic conditions and living standards are higher, while the family size is smaller as compared to village. The environments, diets and lifestyles in urban areas are different from the rural places and moreover, the use of antibiotics in baby from city may be more frequent than in village, therefore, these factors would affect the “healthy” microbiota which is benefit for the human body and increase the chance to expose the indoor allergens, resulting in a higher risk of AR.

An increased risk of allergic diseases among individuals who are exposed to tobacco smoke has been reported [17]. Our results also confirm smoking history is a risk factor associated with AR. Smoking may facilitate sensitization to both perennial indoor and some outdoor allergens [18]. Furthermore, smoking augments nasal response to allergens in AR patients showing a high IgE, IgG4, and histamine levels in nasal lavage [19]. Smoking in China is prevalent, as China is the largest consumer and producer of tobacco in the world. Increase of smoking people might be one of the plausible explanations for the increase of AR prevalence.

One interesting finding is that the use of computer may pose a risk to AR incidence. Since the computer is not cleaned frequently, dust, dirt, hair, and other debris can build up on the surface of the hardware and also accumulate on the surface of the monitor, where this is a favorite environment for dust mites. Use of computer will increase the chance to contact the allergens and may be an important risk for sensitizations in patients with AR. Although the trend of larger AR proportion in the group of higher frequency of computer use (>5 hs per day) existed (S1 Figure), we failed to confirm its association with the risk of AR by using logistic regression model (**Table 1**). This would be mainly due to a smaller sample size in the groups of using computer 5–8 hs (n = 149) and more than 8 hs (n = 28) as compared to the group of never using computer (n = 5,147) (S1 Figure). In addition, there were some other confounding factors which we could not control, such as the information about the percentages of people owing a computer in rural or urban areas, the information about the use of a desktop or laptop computer, and the amount of time spending indoor.

Home renovation is also a risk factor associated with AR. Formaldehyde is the major air pollutant when making interior decoration and can irritate nasal mucosa, resulting in airway inflammation. Pet ownership has been increased in the urban city in China and our results showed that exposure to pets increase slightly the risk of AR. Pet fur and dander are the sourced materials of pet allergens.

In conclusion, the present study showed that in Guangzhou city, the AR prevalence was significantly higher in the urban area than the rural areas, intermittent AR was the predominant type, and a high proportion of AR patients was clinically undiagnosed or without specific medical therapy. Public health policies should optimized the medical resources and help the patients benefit from a proper diagnosis and treatment. The results also demonstrated a spectrum of AR trigger factors and risk factors and indicated that other than genetic aspect, change of living environment and lifestyles would pose potential risks for AR incidence and exacerbation of symptoms. Strategies are required to monitor and reduce the exposure to risk factors. The epidemiological information will benefit the public health policy maker to design some guideline or medical scheme on the prevention and treatment of AR in Guangzhou, and even in other metropolitan cities in China.

## Supporting Information

S1 Figure **Proportion of the AR prevalence in the groups of computer usage.** The trend of the AR prevalence was analyzed by Chi-Square test. A *p*-value below 0.05 was considered statistically significant.
(TIFF)

## References

[1] Zhang L, Han D, Huang D, Wu Y, Dong Z, et al (2007) Prevalence of self-reported allergic rhinitis in eleven major cities in china. Int Arch Allergy Immunol 149:47–57. 10.1159/000176306 19033732

[2] Asher MI, Keil U, Anderson HR, Beasley R, Crane J, et al (1995) International Study of Asthma and Allergies in Childhood (ISAAC): rationale and methods. Eur Respir J 8:483–491. 7789502 10.1183/09031936.95.08030483

[3] Bousquet J, Van Cauwenberge P, Khaltaev N, Aria Workshop Group WHO (2001) Allergic rhinitis and its impact on asthma. J Allergy Clin Immunol 108:S147–334. 11707753 10.1067/mai.2001.118891

[4] Sly RM (1999) Changing prevalence of allergic rhinitis and asthma. Ann Allergy Asthma Immunol 82:233–248. 10094214 10.1016/S1081-1206(10)62603-8

[5] Kusunoki T, Morimoto T, Nishikomori R, Yasumi T, Heike T, et al (2009) Changing prevalence and severity of childhood allergic diseases in kyoto, Japan, from 1996 to 2006. Allergol Int 58:543–548. 19700935 10.2332/allergolint.09-OA-0085

[6] Li J, Sun B, Huang Y, Lin X, Zhao D, et al (2009) A multicentre study assessing the prevalence of sensitizations in patients with asthma and/or rhinitis in China. Allergy 64:1083–1092. 19210346 10.1111/j.1398-9995.2009.01967.x

[7] Wang JH, Devalia JL, Duddle JM, Hamilton SA, Davies RJ (1995) Effect of six-hour exposure to nitrogen dioxide on early-phase nasal response to allergen challenge in patients with a history of seasonal allergic rhinitis. J Allergy Clin Immunol 96:669–676. 7499684 10.1016/s0091-6749(95)70266-0

[8] Liu MM, Wang D, Zhao Y, Liu YQ, Huang MM, et al (2013) Effects of outdoor and indoor air pollution on respiratory health of Chinese children from 50 kindergartens. J Epidemiol 23:280–287. 23728483 10.2188/jea.JE20120175 PMC3709542

[9] Lee SY, Chang YS, Cho SH (2013) Allergic diseases and air pollution. Asia Pac Allergy 3:145–154. 23956961 10.5415/apallergy.2013.3.3.145 PMC3736369

[10] Bauchau V, Durham SR (2004) Prevalence and rate of diagnosis of allergic rhinitis in Europe. Eur Respir J 24:758–764. 15516669 10.1183/09031936.04.00013904

[11] Li J, Huang Y, Lin X, Zhao D, Tan G, et al (2012) Factors associated with allergen sensitizations in patients with asthma and/or rhinitis in China. Am J Rhinol Allergy 26:85–91. 22369791 10.2500/ajra.2012.26.3751

[12] Dávila I, Mullol J, Ferrer M, Bartra J, del Cuvillo A, et al (2009) Genetic aspects of allergic rhinitis. J Investig Allergol Clin Immunol 19:25–31. 19476051

[13] Tang XF, Tang HY, Sun LD, Xiao FL, Zhang Z, et al (2012) Genetic variant rs4982958 at 14q11.2 is associated with allergic rhinitis in a Chinese Han population running title: 14q11.2 is a susceptibility locus for allergic rhinitis. J Investig Allergol Clin Immunol 22:55–62. 22448455

[14] Leynaert B, Neukirch C, Jarvis D, Chinn S, Burney P, et al (2001) Does living on a farm during childhood protect against asthma, allergic rhinitis, and atopy in adulthood? Am J Respir Crit Care Med 164:1829–1834. 11734431 10.1164/ajrccm.164.10.2103137

[15] Strachan DP (1989) Hay fever, hygiene, and household size. BMJ 299:1259–1260. 2513902 10.1136/bmj.299.6710.1259 PMC1838109

[16] Sheikh A, Strachan DP (2004) The hygiene theory: fact or fiction? Curr Opin Otolaryngol Head Neck Surg 12:232–236. 15167035 10.1097/01.moo.0000122311.13359.30

[17] Saulyte J, Regueira C, Montes-Martínez A, Khudyakov P, Takkouche B (2014) Active or passive exposure to tobacco smoking and allergic rhinitis, allergic dermatitis, and food allergy in adults and children: a systematic review and meta-analysis. PLoS Med 11:e1001611. 24618794 10.1371/journal.pmed.1001611 PMC3949681

[18] Lannerö E, Wickman M, van Hage M, Bergström A, Pershagen G, et al (2008) Exposure to environmental tobacco smoke and sensitisation in children. Thorax 63:172–176. 18089631 10.1136/thx.2007.079053

[19] Diaz-Sanchez D, Rumold R, Gong H Jr (2006) Challenge with environmental tobacco smoke exacerbates allergic airway disease in human beings. J Allergy Clin Immunol 118:441–446. 16890770 10.1016/j.jaci.2006.04.047

