# Supplementary figures and images for: Epidemiological Characterization and Risk Factors of Allergic Rhinitis in the General Population in Guangzhou City in China
Source: PLoS One. 2014 Dec 16;9(12):e114950. doi: 10.1371/journal.pone.0114950 (PMC4267734; doi:10.1371/journal.pone.0114950)

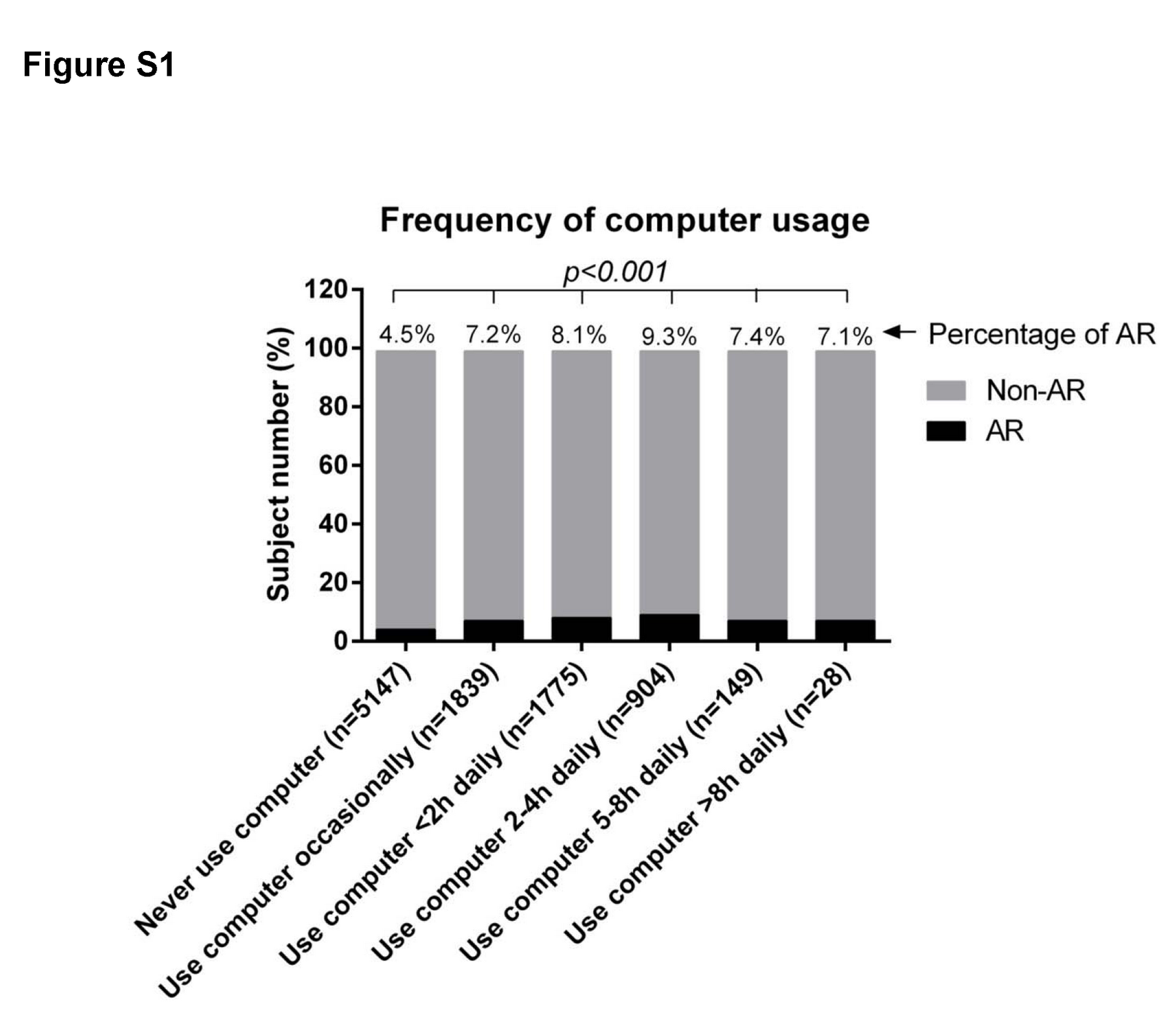

Supplement: S1 Figure — Proportion of the AR prevalence in the groups of computer usage. The trend of the AR prevalence was analyzed by Chi-Square test. A p-value below 0.05 was considered statistically significant. (TIFF) [file pone.0114950.s001.tiff]
